# *Carica papaya* Leaf Juice for Dengue: A Scoping Review

**DOI:** 10.3390/nu14081584

**Published:** 2022-04-11

**Authors:** Bee Ping Teh, Norzahirah Binti Ahmad, Saharuddin Bin Mohamad, Terence Yew Chin Tan, Mohd Ridzuan Bin Mohd Abd Razak, Adlin Binti Afzan, Ami Fazlin Binti Syed Mohamed

**Affiliations:** 1 Herbal Medicine Research Centre, Institute for Medical Research, National Institutes of Health, Ministry of Health Malaysia, Shah Alam 40170, Malaysia; norzahirah.a@moh.gov.my (N.B.A.); terencetyc@moh.gov.my (T.Y.C.T.); ridzuan.ar@moh.gov.my (M.R.B.M.A.R.); adlinafzan@moh.gov.my (A.B.A.); ami@moh.gov.my (A.F.B.S.M.); 2 Institute of Biological Sciences, Faculty of Science, Universiti Malaya, Kuala Lumpur 50603, Malaysia; 3 Centre of Research in Systems Biology, Structural Bioinformatics and Human Digital Imaging (CRYSTAL), Universiti Malaya, Kuala Lumpur 50603, Malaysia

**Keywords:** *Carica papaya*, papaya, leaf, juice, dengue, platelet, phenolic, flavonoid

## Abstract

The potential therapeutic effect of *Carica papaya* leaf juice has attracted wide interest from the public and scientists in relieving dengue related manifestations. Currently, there is a lack of evaluated evidence on its juice form. Therefore, this scoping review aims to critically appraise the available scientific evidence related to the efficacy of *C. papaya* leaf juice in dengue. A systematic search was performed using predetermined keywords on two electronic databases (PubMed and Google Scholar). Searched results were identified, screened and appraised to establish the association between *C. papaya* and alleviating dengue associated conditions. A total of 28 articles (ethnobotanical information: three, in vitro studies: three, ex vivo studies: one, in vivo study: 13, clinical studies: 10) were included for descriptive analysis, which covered study characteristics, juice preparation/formulations, study outcomes, and toxicity findings. Other than larvicidal activity, this review also reveals two medicinal potentials of *C. papaya* leaf juice on dengue infection, namely anti-thrombocytopenic and immunomodulatory effects. *C. papaya* leaf juice has the potential to be a new drug candidate against dengue disease safely and effectively.

## 1. Introduction

The use of medicinal plants in disease prevention and treatment has been around for many generations worldwide and some of them have been scientifically proven. Various medicinal plants, especially those with antiviral activity have drawn interest from researchers to formulate new medicinal drugs for infectious diseases around the world [1]. Like other plants, *Carica papaya* L. has a high content of phytochemicals that not only have beneficial food nutritional values but also medicinal potential. Its leaves contain alkaloids [2,3,4,5], flavonoids [3,6,7,8], phenolic acids [6,7,8], saponin [9], amino acids [6], organic acids [6], vitamins [8,10], minerals [10], carbohydrates [6] and carotenoids [8]. Traditionally, this plant is used to lower blood pressure and blood sugar levels by ingesting boiled young leaves [11]. Infusion or decoction of the leaves taken orally not only regulate blood pressure but can also treat overweight condition. This plant is very famous for its edible fruit to relieve constipation. Its seeds can be pounded and then ingested to treat intestinal worms. Toothache, corns and warts can be treated by topically applying its sap [12]. Recently, the *C. papaya*, particularly its leaf part, has attracted wide attention for its potential use in dengue treatment.

Dengue, an *Aedes* mosquito-borne viral infection, has become a public health risk that demands the world’s focus, especially in tropical countries [13]. This infection is one of the crucial health concerns in Malaysia because of its worrying statistical value. There were more than 900,000 dengue cases with over a thousand deaths reported in Malaysia from the year 2009 to present and more than 125,000 dengue cases were reported in 2019 alone [14]. The dengue virus (DENV) belongs to the virus family *Flaviviridae* and consists of five serotypes (DENV-1 to 5). Compared to other serotypes, the fifth serotype has only been circulated between non-human primates and mosquitoes (also known as the sylvatic cycle) with only one new human infected case reported in 2013 [15,16]. The ideal vector for DENV-5 is mosquito *Aedes niveus*, whilst the female *Aedes aegypti* mosquito is the common vector in DENV transmission [16,17]. Compared to the male type, the female mosquito requires external blood as a nutrient supply, particularly iron mineral, for its eggs production and development [18]. Due to the very low transmission rate of the DENV-5 and a lack of scientific information, at present the World Health Organization upholds that the dengue disease is a public health concern mainly caused by the other four serotypes [19].

There are primary and secondary dengue infections. Individuals who have recovered from primary infection of a serotype have lifelong immunity against the same serotype but the risk of developing severe dengue would be higher following infection by any other of the three serotypes (secondary infection) [20,21]. Severe dengue, also known as dengue hemorrhagic fever, is potentially fatal. Despite of the life-threatening complication, there are still no specific antiviral medications for dengue infection [22]. Nevertheless, scientists are persistently striving to find the cure by diverging their attentions to develop a therapeutic drug that can disrupt or cease targeted DENV proteins’ functions [23]. A licensed dengue vaccine commercially available in some countries is not effective against all four common serotypes of DENV, and it can cause individuals unexposed to DENV to be at greater risk of developing severe disease [24,25]. These situations highlight the research need in identifying potent compounds with promising anti-dengue activities via in depth understanding of dengue pathogenesis. There are currently no systematic scoping reviews focused on *C. papaya* leaf juice. Therefore, this scoping review was conducted to gather and highlight the available scientific evidence for the use of *C. papaya* leaf, particularly using juice form in treating dengue infection.

## 2. Materials and Methods

This scoping review was designed based on framework adapted from Arksey and O’Malley’s study (2005) [26]. A systematic search was conducted by two independent investigators using combination of keywords on PubMed and Google Scholar electronic databases. The keywords used at PubMed were ‘carica papaya’ AND (‘leaf’ OR ‘leaves’) AND ‘juice’ AND ‘dengue’ used in all fields. While the search setting used in Google Scholar was: (1) with exact phrase of ‘carica papaya’; (2) with all the words of ‘leaf, ‘leaves’, ‘juice’, and ‘dengue’; and (3) include citations and anywhere in the article. The search period was in the default setting for both electronic databases until 1 March 2022. The search result was manually screened and selection of the included articles was limited to: (1) English language journal article; (2) full text accessibility; (3) conducted study on the juice derived from *C. papaya* leaf; (4) related to anti-dengue activity; and (5) article about ethnobotanical information, in vitro, in vivo and human studies. The definition of juice for this review referring to any liquid form originated from the *C. papaya* by crushing, pounding, pressing, cutting, squeezing and/or blending its leaves. A bibliographic manager (EndNote version 20, Clarivate Analytics, Philadelphia, PA, USA) was used to manage the search results. Data extraction for included studies was performed independently by two authors using a customized data extraction table (Table 1). Any disagreement was reviewed by a third author.

## 3. Results

### 3.1. Study Inclusion

**Figure 1:**
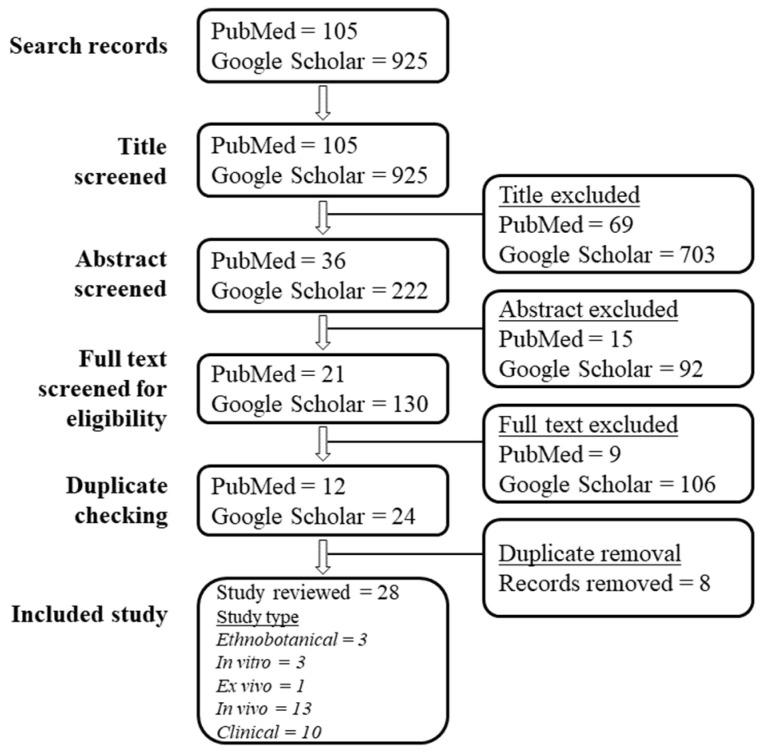
Preferred reporting items for systematic review and meta-analysis (PRISMA) chart of articles searching and screening. Note: One included study conducted in vitro, ex vivo and in vivo experiments, which resulted in an additional three types of study.

From a total of 1030 records identified from keyword searches on the selected online databases, a final 28 articles were included in this scoping review, as presented in the preferred reporting items for systematic review and meta-analysis (PRISMA) chart (Figure 1).

### 3.2. Study Characteristics

The 28 articles included in the review reported ethnobotanical information (*n* = 3), in vitro findings (*n* = 3), ex vivo findings (*n* = 1), in vivo findings (*n* = 13) and clinical findings (*n* = 10). These findings are summarized in Table 2. The three surveys compiling ethnobotanical information were in the regions of Bangladesh and the Philippines [27,28,29]. The three included in vitro studies used blood cells from human subjects and laboratory rats [30,31,32], whilst the only ex vivo study used bone marrow cells and splenocytes isolated from laboratory rats [32]. Among the 13 included in vivo studies, one study used mosquitoes [33], six studies used rat model [32,34,35,36,37,38] and seven studies used mouse model [38,39,40,41,42,43,44].

Among the 10 included clinical studies, three studies are case reports [45,46,47], one cross-sectional study [48], four quasi trials [49,50,51,52] and two open-labelled randomized controlled trials [53,54].

### 3.3. Interventions Used

The processing method is critically correlated to the phytochemical contents and concentrations of a medicinal plant, different content and concentration can later affect the efficacy of the plant. Several juice preparation methods, including maturity of leaf and leaf cleaning details, of the included studies were highlighted (Table 3). Several included studies highlighted the leaf maturity used in their experiments [28,30,31,32,34,36,39,44,47,49,52,54] and one of these studies found that three different leaf maturities had similar content of phytochemicals such as flavonoid [30]. The potential of *C. papaya* leaf juice for anti-dengue activities is associated with its phytochemicals (Table 4). Among the 28 included studies, only several of them investigated the chemical profiles of the leaf juice used. There is one sub class of phenolics commonly identified in the 10 included studies, i.e., flavonoids [30,32,34,37,38,40,41,42,43,53]. The flavonoids detected were namely flavones (myricetin) and flavonols (quercetin analogue (clitorin, rutin) and kaempferol analogue (manghaslin, nicotiflorin)) were the most abundant flavonoid contents identified in the leaf juice [38,40,41,42,43].

Only one study investigated the effect of using male and female *C. papaya* plants [39] and another one highlighted the exact variety of *C. papaya* used [53]. Due to the bitter taste of *C. papaya* leaf juice, two included studies added sucrose into the juice preparation [46,52] whilst another two studies allowed the consumption of leaf juice with another liquid-based edible ingredient (milk and commercially-made fruit juice) [28,45]. However, one study that prescribed one kiwi fruit along with *C. papaya* leaf juice intervention suggested the relief symptoms of muscle pain and skin rashes were possibly due to the kiwi fruit [51].

Seven out of the 10 clinical studies allowed conventional treatment (such as fluid replacement, antipyretics, antibiotics, antimalarial drugs, immunosuppressant and antiemetics) for the dengue patients on top of the intervention treatment. Briefly, two studies treated the patients using conventional treatment before starting the intervention treatment [45,46]. Four studies allowed conventional treatment together with intervention treatment [47,48,49,53], whilst only one study treated the patients using conventional treatment after completion of intervention treatment period [52]. Only one study used a combination of the intervention, Ayurveda therapy and conventional treatment [47].

### 3.4. Ethnobotanical Findings

Traditionally, limited published material has reported the use of *C. papaya* leaf juice in dengue treatment. Out of the 28 included studies, only three showed evidence of traditional use of interest. All three articles are collective survey studies, which involved 92 local residents (the majority were traditional health practitioners) in the selected regions of Bangladesh [27] and the Philippines [28,29]. However, certain information cannot be found from all three articles, such as the amount of leaf juice being used, juice preparation and consumption period (Table 2 and Table 3).

### 3.5. In Vitro Findings

Three included studies investigated the effect of *C. papaya* leaf juice at different maturity using cells isolated from healthy and dengue patients and healthy rodents, respectively (Table 2). According to Ranasinghe et al., (2012), no significant difference was found on hemolysis inhibition level (*p* > 0.05) between three different maturities of leaves on both types of heat-induced hemolysis erythrocytes (healthy: 31.8–38.5%, dengue: 25.7–32.5%), respectively, compared to the aspirin control group (healthy: 45.9%, dengue: 43.6%). Testing on hypotonicity-induced hemolysis erythrocytes showed the inhibition level was also not significantly different (*p* > 0.05) in the intervention-treated group (partly mature leaves) on both types of erythrocytes (healthy: 31.57%, dengue: 57.03%) compared to the indomethacin control group (healthy: 47.67%, dengue: 65.35%) [30].

Chinnappan et al., (2016) investigated the efficacy of *C. papaya* leaf juice on adenosine diphosphate-induced platelet aggregation on plasma rich platelets and plasma poor platelets obtained from healthy volunteers and dengue patients. They found that platelet aggregation was significantly lower (*p* < 0.05) in both intervention-treated healthy and dengue plasma rich platelets than the untreated group. The same observation occurred (*p* < 0.05) for intervention-treated healthy plasma rich platelets (which was pre-infected with intervention pre-treated dengue plasma poor platelets) compared to the untreated group [31].

Phagocytic activity in intervention-treated peritoneal macrophages (62.5–1000 µg/mL) significantly increased (*p* < 0.05) by 72.91–189.58% (non-dose dependent manner) compared to media control group while levels of interferon (IFN)-γ and interleukin (IL)-10 significantly increased (*p* < 0.05) in dose dependent manner, compared to the media control group [32].

Beneficial uses of *C. papaya* leaf juice on key clinical manifestations in dengue infection were shown by in vitro findings through the inhibition of hemolysis erythrocytes was comparable with aspirin and indomethacin drugs in stabilizing the plasma membrane of dengue patients. Therefore, the risk of patients having plasma leakage due to increased vascular permeability and capillary fragility can be reduced. The DENV non-structural protein 1 was not only able to induce platelet activation but also aggregation through toll-like receptor 4 [55]. Interestingly, the leaf juice was found to be able to inhibit the excessive aggregation of platelets, which suggests thrombocytopenia and hemorrhage conditions triggered by dengue infection can be minimized. Effect on phagocytic activity and cytokine release also provide an insight on the potential of *C. papaya* leaf juice in modulating functional and non-functional immune responses which are triggered when a pathogen invades the host’s body.

### 3.6. Ex Vivo and In Vivo Findings

There are 13 included studies using mouse and rat models, whilst there is only one study using mosquitoes to investigate the potential use of *C. papaya* leaf juice as a method to combat dengue (Table 2). Vector control via the use of larvicidal is a straightforward way of controlling mosquito vector borne diseases. The common vector for dengue is *Aedes aegypti* mosquitoes and it has four life stages (egg, larvae, pupa and adult). It only takes up to 10 days for the eggs to evolve into adults [56]. Therefore, it is critical to stop the emergence of the adult mosquitoes as early as possible. However, from the article search targeting only juice form, the *C. papaya* leaf juice was found to have an insecticidal effect on the larvae in one study. Rubio (2016) found that mosquito larvae such as *Aedes* sp. died 5 to 30 minutes after the addition of the leaf juice into the artificial trap containing clean water [33].

Apart from an in vitro study, Jayasinghe et al., (2017) also studied the effect of leaf juice on healthy Wistar rats. The team used bone marrow cells (absence of mitogen) and splenocytes isolated from healthy Wistar rats. Both cells significantly proliferated (*p* < 0.01, by 63.2% and 39.62%) in the intervention-treated group (only 31.25 µg/mL). Level of IFN-γ from splenocytes (31.25–1000 µg/mL) significantly increased (*p* < 0.05) in intervention groups whilst the level of IFN-γ from bone marrow cells only increased starting from treatment level of 62.5 µg/mL. Level of IL-10 from bone marrow cells (only at 62.5 µg/mL) and the IL-10 from splenocytes (31.25–500 µg/mL) significantly (*p* < 0.05) increased in intervention groups. After three days of oral treatment, platelet count (increased by 68%), bone marrow cells (49%) and total white blood counts (19%), monocytes (44.67%), lymphocytes (10%), pro-inflammatory cytokines (tumor necrosis factor (TNF)-alpha (39.09%), IL-6 (55.06%)) and phagocytic activity (109%) in the intervention-treated group had promising results (*p* < 0.05) [32].

Compared to the procedures used in Jayasinghe et al.’s study, one study isolated the cells only after treating the healthy Wistar rats with leaf juice for three days. Platelet counts (increased by 68%), differential white blood cells (monocytes: 44.67%; lymphocytes: 10%) and bone marrow cells (35%) in the intervention-treated group were better than in the distilled water-control group (*p* < 0.05). Similarly, phagocytic activity of peritoneal macrophages was also measured, and it significantly increased (*p* < 0.05) in the intervention-treated group (109%) compared to distilled water-control group [34].

Out of the 12 studies using a rodent model, there were four studies using a thrombocytopenia rat model (Table 2). Akhter and team (2015) found that after three days of treatment, platelet counts significantly increased (*p* < 0.05) in the intervention-treated group (7.83 × 10^5^/µL) compared to the hydrocortisone control group (4.05 × 10^5^/µL) [35]. Pure leaf juice administration (0.18 mL/100 g body weight, for five days) significantly increased (*p* < 0.05) the red blood count on the fifth day of treatment compared to the untreated control group. Bleeding time was significantly shorter (*p* < 0.05) in the intervention-treated groups on the second and fifth day of treatment compared to the untreated control group [36].

It has been reported that there was a dose-dependent efficacy of the leaf juice when compared to the untreated control group after 14 days of treatment. Bleeding and clotting times were significantly shorter (*p* < 0.05) in the intervention-treated groups since the eighth day of treatment. Levels of cellular malondialdehyde (*p* < 0.001) and serum thrombopoietin cytokine (*p* < 0.05) were downregulated whilst levels of cellular antioxidant enzyme (SOD) and GSH (*p* < 0.001) were upregulated in the intervention-treated groups on the 14th day of treatment. In contrast, platelet count significantly increased (*p* < 0.001) in the intervention-treated groups starting from the eighth day of treatment when compared to the water control group. Without a noticeable dose dependent effect, promising findings such as shorter prothrombin times and higher expression level of MPL-CD110 receptor (*p* < 0.05) were observed in the intervention-treated group on the 14th day of treatment compared to the untreated control group. Mature megakaryocytes with high cellularity and erythroblast cells in bone marrow were observed in the intervention-treated group compared to the untreated group [37].

Using a similar animal model and same time length of intervention administration, Anjum et al., (2017) found that not only did the platelet counts increase (*p* < 0.01) starting from day seven of treatment in the intervention-treated groups, but the levels of monocytes, basophils, eosinophils, lymphocytes and neutrophils also increased (*p* < 0.01) in the intervention-treated groups (after 14 days of treatment), compared to the saline control group. Clotting and bleeding time were significantly shorter (*p* < 0.01) in the intervention-treated groups compared to the saline control group [38].

Apart from using rats (Table 2), Anjum et al., (2017) also administered the same juice preparation to thrombocytopenic mouse model (the condition was re-induced on the fifth to seventh day after three days of intervention treatment) to investigate immunomodulatory activity against the untreated control group. Inflammation level was significantly lower (*p* < 0.01) in the intervention-treated group after 48-h of treatment. Total leukocyte counts were significantly higher (*p* < 0.05) in the intervention-treated group. Phagocytic index, mean antibody titer and level of TNF-α (a pro-inflammatory cytokine) were significantly lower (*p* < 0.05) in the intervention-treated group [38]. Myelosuppression mice were administered with the leaf juice at two different doses (5 and 10 mL/kg body weight), respectively, for 21 days. There was no significant difference (*p* ≥ 0.005) on platelet count increase from day 7 to 21 between male (719–950 × 10^9^/L) and female (700–979 × 10^9^/L) plant variety of both doses of the intervention-treated groups. Platelet count significantly increased (*p* < 0.001) in the intervention-treated groups (in a dose dependent manner) starting from day seven (700–793 × 10^9^/L) compared to the distilled water-control group (78 × 10^9^/L) [39].

Mohd Ridzuan and teams (2018–2021) used mouse model of DENV-infection (also known as AG129 mouse) in their four studies to investigate the therapeutic effect of leaf juice with a three-day treatment period. Three studies used New Guinea strain-DENV-2 and another one used the Malaysian clinical DENV-2 strain. In their first study, no significant difference (*p* > 0.05) on plasma antigen level was found between the intervention-treated group (1000 mg/kg body weight) on the last day of intervention treatment as well as on second and fourth day after the treatment period, compared to the distilled water group. Similar findings were reported for the plasma viral RNA of intervention-treated group on the second and fourth day after the treatment period [40]. Subsequently, they reported that the same preparation affected four gene expressions (CCL2, ITGB3, FN1, ICAM1) of endothelial cell biology in the liver of the intervention-treated group compared to the untreated group [41]. Their teams also found that the expressions of eight genes (CCL6/MRP-1, CCL8/MCP-2, CCL12/MCP-5, CCL17/TARC, IL1R1, IL1RN/IL1Ra, NAMPT/PBEF1 and PF4/CXCL4) were downregulated (*p* < 0.05) a day after the treatment period compared to the untreated group [42].

Based on their latest published study, the same leaf juice preparation on mice infected with a different strain of DENV-2 provided some other findings. Total white blood cell count and neutrophils in the intervention-treated group (1000 mg/kg body weight) significantly increased (*p* < 0.05) by 1.44-fold compared to the untreated group. Five plasma cytokines (GM-CSF, GRO-α, IL-6, MCP-1, MIP-1β) significantly decreased (*p* < 0.05) in the intervention-treated group (both doses) compared to the untreated group. Whereas levels of IL-1β (as plasma cytokine) and IL-6 (as intracellular cytokine expression in the liver) were only significantly lower (*p* < 0.05) for 500 mg/kg for the intervention-treated group compared to the untreated group. Dengue viral RNA level in the liver was significantly lower (*p* < 0.05) in intervention-treated group (1000 mg/kg body weight) compared to the untreated group [43]. Out of seven included in vivo studies using a mouse model, only one study investigated the efficacy of *C. papaya* leaf juice (seven-day administration) on healthy mice. Levels of platelet and red blood cells were significantly higher (*p* < 0.05) starting from a day after the treatment period in the intervention-treated group compared to the water control group [44].

Developing a safe and effective antiviral drug is challenging because viruses use host’s cells in their replication. The pathogenesis and severity of dengue are linked to immune response interruption caused by the DENV. The DENV not only suppresses bone marrow level, but also attacks by binding to the platelets, which are tiny disc-shaped cells that are produced by megakaryocytes (mature white blood cells) in bone marrow before entering the blood bloodstream and spleen. The generated antibodies for these DENV will then identify and flag the infected-platelets as foreign matter to be exposed to the host’s body lines of defense. Infected vascular endothelial cells will have platelets aggregated in them and the generated antibodies eventually kill these cells and platelets too. Such an immune response certainly reduces the platelet counts in the dengue patients. Whilst the bone marrow suppression would lead to anemia and hemorrhagic events [57,58]. Therefore, a drug that protects bone marrow, stimulates platelet production and shortens bleeding and clotting time would be ideal.

Evaluated in vivo evidence in this review mostly induced a thrombocytopenia condition in the animal model and several experiments using a dengue specific animal model to mimic the dengue’s key clinical manifestations as much as possible to provide better confidence in the research findings. A gene expression profile of inflammatory cytokines and receptors found to be associated with the presence of dengue infection. Interestingly, the degree of liver damage has been proposed to be correlated to DENV infection because the liver is surrounded with sinusoidal endothelium and during the DENV invasion, endothelial permeability increases. Consequently, irregular levels of liver function enzymes, histopathological lesion and traces of dengue antigen can be detected in liver tissue. Both dengue infected and healthy animal models used in the included studies imply the pharmacological role of the *C. papaya* leaf juice on functional (activation of phagocytosis and cell proliferation) and non-functional (regulation of immune cell level and release of inflammatory cytokines) immune responses.

### 3.7. Clinical Findings

Patients were confirmed with dengue infection after checking their blood parameters for the presence of dengue immunoglobulin G antibody, dengue immunoglobulin M antibody, and/or non-structural protein 1 antigen [45,47,48,49,51,52,53,54], whilst some patients were diagnosed without any antigen or antibody test but solely based on low platelet counts (not more than 150,000/µL) and clinical symptoms such as high fever and body ache [46,50].

#### 3.7.1. Case Reports

The different juice preparations used were documented in three case reports (Table 2). These dengue patients, aged 23–51 years old, were also receiving standard treatment associated with dengue symptoms such as fluid replacement, antipyretics, antibiotics, antimalarial drugs, immunosuppressant and antiemetics [45,46,47]. With no symptom improving after receiving five-day standard treatment in the ward, a male patient started to consume the intervention (150 mL) once daily with alternative sip of commercially made fruit juice for the next five days of hospitalization. The platelet count showed some progress (increased by 1.10 × 10^5^/µL), as did the level of white blood cells (by 4.8 × 10^4^/µL) and level of hemoglobin (by 0.5 g/µL) [45]. With the same five-day duration of intervention treatment but consumption frequency increased to twice daily, Ahmad et al., (2011) found that the intervention (25 mL) upregulated the levels of platelets, white blood cells and neutrophil in the male patient starting on the second day of treatment and returned to a normal range on the fifth day of treatment compared to the starting the intervention after five days of standard treatment [46]. Along with receiving standard treatment together with the intervention (25 mL twice daily) for five days, this male patient also received Ayurveda treatment for a further three days together with the intervention. The platelet counts (increased by 1.73 × 10^5^/µL) and white blood cell counts (by 7.3 × 10^4^/µL) showed a good sign of recovering. The patient was discharged after completing the eight-day intervention treatment period [47].

#### 3.7.2. Cross-Sectional Study

A cross-sectional study (Table 2) involving 214 dengue patients aged ≥18 years old who were admitted to Hospital Universiti Sains Malaysia between January 2014 and December 2015 was conducted to collect information on the use of traditional and complementary medicines (T&CM), which they believe can treat dengue. There were 131 respondents that consumed *C. papaya* leaf juice at least once daily for three days while receiving standard treatment throughout their hospitalization. Another two T&CMs reported to be commonly used were crab soup (174 respondents) and isotonic drinks (126 respondents) [48].

#### 3.7.3. Quasi Experiment

Comparing before and after treatment effect (Table 2), Hettige (2008) investigated the efficacy of *C. papaya* leaf juice both adult dengue patients (eight subjects aged 12–55 years old, three females and five males) and children (four subjects aged 5–8 years old, three females and one male), who also received standard oral treatment. After a one-day intervention treatment period, their platelet counts increased by 3000–58,000/µL and white blood cell counts increased by 350–3400/µL. Five of the patients no longer experienced a hemorrhagic skin rash after consuming the intervention. All 12 patients recovered with no hospital admission [49]. Prakash (2012) found that platelet count of all five dengue patients (19–52 years old) increased by 8000–11000/µL after consuming the leaf juice (two tablespoons, three times for one day) [50].

Solanki et al., (2020) studied a larger sample (100 dengue patients: 42 females and 58 males in the intervention group vs. 50 patients: 20 females and 30 males in the control group) who received the leaf juice treatment (adult: 10 mL, children: 5 mL) thrice daily for three days together with one kiwi fruit. Platelet count in the intervention-treated group significantly increased (*p* < 0.05) by 6.31 × 10^5^/µL, whilst it only increased by 2.64 × 10^3^/µL in the control group, compared to before receiving treatment. Compared to the levels before treatment, the increase in white blood cells (*p* < 0.05) was higher in the intervention-treated group (1.03 × 10^3^/µL) than the control group (1.73 × 10^2^/µL) [51]. In a clinical study investigating the effect of a six-day leaf juice treatment (5 mL thrice daily), it was reported that not only did platelets and total white blood cell counts increase (*p* < 0.05) on day 6, but also all nine dengue patients (six females and three males) recovered from lethargy, fatigue and fever [52].

#### 3.7.4. Randomized Controlled Trial

Two open-labelled randomized controlled trials were included in this review (Table 2). Subenthiran et al., (2013) assessed the platelet count in 18–60 years old dengue patients (111 patients: 20 females and 91 males in the intervention group vs. 117 patients: 14 females and 103 males in control group) after consuming 30 mL of *C. papaya* leaf juice for three days as well as receiving standard treatment. Compared to 8-h hospitalization, the platelet count after 40-h hospitalization was higher (*p* = 0.019) (mean difference = −7.890) whilst the platelet count in the control group had a significantly higher value (−7.703) only after 48-h hospitalization. Arachidonate 12-lipoxygenase (ALOX12) and platelet-activating factor receptor (PTAFR) genes highly expressed in intervention-treated groups, respectively, (ΔCt mean = 16.02, fold change = 15.00; ΔCt mean = 14.87, fold change = 13.42) compared to control group [53]. Another similar trial reported significant findings on other outcome parameters in 16–60 years old in-ward dengue patients (43 patients: 7 females and 36 males in the intervention group vs. 76 patients: 15 females and 61 males in the control group) that consumed 20 mL of the leaf juice twice daily until discharged. The total duration of illness, fever and hospitalization was significantly shorter (*p* < 0.05) than the control group. Episodes of pleural effusion were also lesser (*p* < 0.05) in the intervention-treated group (two subjects) compared to the control group (12 subjects) [54].

Dengue hemorrhagic fever is a potentially life-threatening complication found among dengue patients. Clinically, two common severe dengue manifestations are a rapid drop of platelet count and severe hemorrhage (caused by plasma leakage). The mechanism underlying these phenomena correlate to the devastation of infected-platelets and bone marrow suppression during DENV invasion, as mentioned earlier. Certain genes can regulate different biological processes, such as ALOX12 and PTAFR. The ALOX12 gene is highly expressed in platelet, megakargocytes and epidermis, and able to produce 12(S)-hydroxyeicosatetraenoic acid (HETE), which is an essential inflammatory signaling molecule. Therefore, the ALOX12 gene is involved in regulating platelet activation, cell apoptosis, endothelial cell migration and cell proliferation [59]. Platelet activating factor (PAF) is a phospholipid activator and mediator of white blood cells, platelet aggregation, inflammation and anaphylaxis. A G-protein coupled receptor 1 family binds to the PAF (and then forms PTAFR). Therefore, during virus invasion into body, high expression of the PTAFR genes could indicate an active inflammation response to release more platelets [60]. Gathered clinical evidence in this review clearly shows the likelihood of *C. papaya* in leaf juice form improving the thrombocytopenia condition and modulating the immune response during dengue infection. Apart from these, the leaf juice also seems to be able to relieve dengue symptoms such as fever, skin rash, lethargy, fatigue, pleural effusion, sick period and hospitalization period. Two clinical studies appraised in this review did not recruit dengue patients diagnosed with dengue hemorrhagic fever and/or with an irregular level of liver enzyme and/or low level of creatine kinase. These exclusion criteria were possibly to standardize the severity level of dengue infection among the recruited patients in order to accurately assess the effectiveness of intervention. There are reports that correlated hepatic dysfunction to dengue infection and even proposed the possibility of using the level of liver enzymes as a reference point in predicting severity of dengue infection [61,62,63]. Therefore, not recruiting dengue patients with underlying liver-related problems minimizes the confounding factors that could influence the study outcome.

### 3.8. Safety Findings

Apart from reporting dengue related clinical manifestations, several included studies also shared safety-related observations. Two three-day oral acute toxicity studies using 0.72 mL *C. papaya* leaf juice per 100 g body weight of healthy rats neither showed hepatoxicity or nephrotoxicity [32,34], but a cytotoxicity effect was observed in healthy bone marrow cells and splenocytes treated with 500 and 1000 μg/mL of leaf juice [32]. Nandini et al., (2021) found that healthy rats treated with 5–2000 mg/kg body weight of leaf juice had a significantly lower level of alanine aminotransferase (*p* < 0.05) compared to the untreated control group; however, fewer lesions were found on the liver and kidney in the intervention-treated group [37]. Cyclophosphamide-induced thrombocytopenia rats treated with the leaf juice (50 and 150 mg/kg body weight) had fewer histological changes on their livers and spleens compared to the saline-treated group [38]. In addition, Mohd Ridzuan and his team (2018) found that body weights of both mock infected AG129 mice groups: one group treated with 1000 mg/kg body weight of leaf juice, whilst another group treated with distilled water for three days, remained unchanged until the seventh day after the treatment period [40]. In his later study using AG129 mice treated with 500 and 1000 mg/kg body weight of leaf juice for three days, there was no significant body weight changes (*p* > 0.05) between the intervention-treated and untreated mock infected groups. Spleen size of the mice were also not significantly different (*p* > 0.05) between the intervention-treated dengue infected group and untreated mock infected group [43].

With the current available information on dengue pathogenesis, several organs (such as the liver (as mentioned earlier) and spleen (one site produces platelets)) and cells (such as platelets, bone marrow cells, splenocytes) are found to be the target of infection. Based on the above reported safety findings, treatment of *C. papaya* leaf juice, up to certain doses and treatment duration, seems to have a protective effect on the organs of dengue infected subjects.

## 4. Discussion

In summary, this is the first review on juice form of *C. papaya* leaf consisting of 28 included studies that focused on efficacy of this herbal preparation on dengue related parameters. The doses of juice used and treatment duration were varied. Interestingly, the findings from the included studies seemed to be associated with the use of *C. papaya* leaf juice. Therefore, regardless of variation in dose and treatment duration, this suggests the potential use of the leaf juice in treating dengue manifestation.

This review found that *C. papaya* leaf juice does not demonstrate a linear dose-response relationship in the measured study parameters [32,36,37,38,43]. Nevertheless, s hormesis dose-response relationship was observed where beneficial effects were observed at low doses instead of high dose. Such a dose-response model has been reported to occur in some therapeutic agents such as antiviral drugs [64]. The DENV replication in a host’s body would only be successful by inhibiting the signals of interferon in the body. In conjunction with this factor, a reliable mouse research model in dengue research was established. The AG129 mouse used in dengue research lacks α-, β- and γ-interferon receptors making the introduced DENV unable to communicate with the host’s interferon and therefore the DENV can successfully replicate in the host’s body [65,66]. The use of such a laboratory model could mimic clinical manifestation of human dengue infection. Another team of scientist also discovered the association between endothelial permeability of liver tissue and DENV and suggested that the liver can be the virus replication site [67]. Despite the efforts investigating larvicide(s) in reducing the number of mosquitoes as a disease vector, there was very few studies pertaining to the larvicidal effect of the leaf juice.

The choice of efficacy studies for medicinal plants is commonly determined by the ethnobotanical information. Ethnobotany is a field of study related to traditional knowledge on use of plants such as for medicinal use. This type of valuable information is mostly based on years of belief and observation; and richer in countries with big ethnic diversity as these regions would have more communities, such as indigenous people, who used or tried alternative healthcare treatment, such as using plants to heal health conditions. Several drugs prescribed in conventional medicine originated from naturally occurring substances and are plant-based such as digoxin extracted from *Digitalis lanata* for hearth problem and morphine (from *Papaver somniferum*) for pain control. Despite the credit of ethnobotany in drug discovery and development, consumers should always keep in mind that not all traditional knowledge of medicinal plants has been therapeutically investigated to establish a safe dose and therefore they should be more cautious in using it [68].

Lately, an extensive review about the safe use of different formulations of *C. papaya* leaves highlighted some safety concerns. Apart from the mild gastrointestinal side effects, interaction with co-administered drugs, such as certain hypoglycemic agents, anti-malarial, cardiovascular drugs, and antibiotics, has either increased or decreased the efficacy of the drugs. It revealed that consumption of *C. papaya* leaves (up to 21 days) affected the reproductivity in both male and female animals. The possibility of consuming products containing *C. papaya* leaves which cause dysregulation of liver enzymes and lesions on the liver, was suggested from three in vivo studies with long treatment duration (up to 35 days). However, no liver function-related side effect was reported in the appraised clinical data, except there were two case reports that documented a plausible association between consumption of *C. papaya* leaf extract and irregular level of liver enzymes but the impact from confounding factors, such as any concomitant drug and/or underlying health condition of the patients, was not described in the reports and therefore such association is still questionable [69].

Despite these unfavorable findings reported in the safety review, there are other aspects that shall be addressed too. Firstly, there is no clinical evidence that reports the adverse effect of *C. papaya* leaves on the human reproductive system, either long or short term. Secondly, as compared to a long consumption period, neither hepatoxicity nor other toxic-related effects were found on the animals treated with a single dose of the leaves aqueous extract within a 14-day study duration [70]. In addition, the safety findings from this review imply protective role of *C. papaya* leaf juice on the organs of dengue infected subjects. Thirdly, the type of solvents used in plant extraction is a key factor that may influence the efficacy and/or level of toxicity observed, especially when toxic chemical solvents are used [71,72].

A series of rodent toxicity studies using freeze-dried *C. papaya* leaf juice, from 14 days of single dosing up to 90 days of daily dosing over a range of doses, showed minimal toxicity findings and the no-observed-adverse-level was 2000 mg/kg body weight [70,73,74]. Hence, a human equivalent dose for the 2000 mg/kg body weight by considering a safety factor of 10 is 32.26 mg/kg body weight, which is equivalent to 2.26 g of the freeze-dried *C. papaya* leaf juice taken by a 70 kg human. The treatment regime prescribed in the included RCTs was 5–30 mL of the juice for adult consumption (vs. children: 2.5–5 mL) for one to six days [49,50,51,52,54].

Flavonoids have already been studied for their antiviral effect on human viruses, such as herpes simplex, polio, parainfluenza and respiratory syncytial viruses [75]. Flavonoids derived from plants have been shown to inhibit dengue viral replication where the in vitro inhibitory effect was observed using plaque assay [76]. Flavanones isolated from *Boesenbergia rotunda* (L.) Mansf. Kulturpfl. showed competitive inhibition towards NS3 protease [77]. Using inhibition kinetics study, docking and protease assay, certain flavonoids were found to inhibit one of DENV enzymes non-structural proteins (NS2B/NS3 protease) at an allosteric site [78]. Other than the protease complex, a few studies also showed commercial flavonoids and flavonoids isolated from different plants inhibited another DENV enzyme (NS5-RNA-dependent RNA polymerase) activity [79,80,81,82]. Other quercetin and kaempferol analogues showed potential for inhibition on DENV enzymatic activities. Both structures, respectively, formed multi-hydrogen bonds with amino acid residues, which enhanced the binding strength of the compounds at the target site [83,84,85,86,87].

The ability of flavonoids to interact with the cell membrane surface that protects the lipid bilayer against harmful agents, such as free radicals, has been discovered [88,89]. Therefore, the reported erythrocyte membrane stabilizing effect could correlate to the high content of flavonoids in the *C. papaya* leaf juice. Similarly, scientists also found an association between flavonoid and immunomodulation activity, such as T helper cell differentiation into inflammatory and regulatory cells via mTOR pathway [90]. Interestingly, in this review, the *C. papaya* leaf juice was also found to be modulating the functional and non-functional immune responses.

As one of the biggest phenolic groups [91] and also the most abundant content found in *C. papaya* leaf juice, flavonoids have also been reported to modulate platelet aggregation (a clinical manifestation that could happen to dengue patients) through few pathways such as inhibition of arachidonic acid, suppression of cytoplasmic calcium ion, blockage of degranulation and integrin signaling mediated by αIIbβ3 integrin, inhibition of platelet granule secretion, and inhibition of thromboxane formation [92]. One study shows the protecting role of bone marrow by *C. papaya* leaf extract in regulating protein carbonyl and glutathione contents within bone marrow, less severity of histology lesion found on the lead-induced oxidative damage bone marrow and promotes production of blood cells and platelet in the bone marrow [93]. Similar prominent findings were also reported by the included studies in protecting or enhancing the production of bone marrow cells and splenocytes.

In Malaysia, *C. papaya* trees are planted for food consumption and commercial purposes. Two papaya varieties are popular in Malaysia, i.e., ‘eksotika’ and ‘sekaki’. Both varieties can be commonly found as hermaphrodite and there are slight differences in their physical appearance [94]. Currently, only one metabolite profiling analysis was conducted on the fruit part of both varieties and a distinct metabolite profile was found between the ‘eksotika’ and ‘sekaki’ [95]. Similar extensive profiling on the leaf part to investigate content difference for both varieties is still lacking.

The phytochemicals detected in the *C. papaya* leaf juice, such as the flavonoids, should be given attention to as they could be potential DENV inhibitors. These points of discussion provided insights on the potential of the *C. papaya* leaf found in this review; for example, recovery of platelet count to minimize risk of bleeding among thrombocytopenic dengue patients. Compared to the three plausible mechanisms of action of *C. papaya* leaf on dengue infection (anti-thrombocytopenic effect, immunomodulatory effect and antiviral effect) suggested by Bok et al., (2020) [96], the findings from this review focusing on leaf juice, emphasize the effect of larvicidal activity, anti-thrombocytopenia and immunomodulation.

Based on the gathered scientific evidence in this review, not only the larvicidal effect, but the *C. papaya* leaf juice also has the potential in relieving dengue manifestations (anti-thrombocytopenic effect and immunomodulatory effect), which are preferable for only a short consumption period, such as the short treatment regime in treating dengue patients.

## 5. Review Limitation

The findings from this scoping review are restricted by several factors. Firstly, only English articles with accessibility of full text were included. Secondly, the therapeutic use of *C. papaya* leaf was only limited to juice form. Thirdly, there were insufficient clinical trials that met inclusion criteria. Fourthly, several included studies provided experimental values in graphic form and thus the authors were unable to compare the findings in one study to another study that measured the same parameter. Consequently, the authors were unable to perform a meta-analysis to draw solid conclusion on anti-dengue activity of *C. papaya* leaf juice. However, given the limitations faced in conducting this scoping review, it is unlikely that any missed data would possibly amend the conclusion drawn based on this review due to a clear focus on juice form obtained from only the leaf part and the electronic database search was performed to include citations and anywhere the keywords appeared in the article.

## Figures and Tables

**Table 1:** Template of data to be extracted for the conduct of this scoping review.

1. Study characteristic	Year Author(s) Title
2. Study type	Ethnobotanical In vitro In vivo Human
3. Subject	Description
4. Intervention	Dose Frequency Treatment duration
5. Comparator	Dose Frequency Treatment duration
6. Outcome	Reported findings
7. Safety outcome	Reported findings

**Table 2:** Summary of included studies using *Carica papaya* leaf juice as dengue treatment.

Study Type (Design, if Any)	Author (Year)	Country	Subject	Intervention	Comparator	Outcome
Ethnobotanical information	Fajardo WT et al. (2017) [28]	Philippines	19 herbalists from 11 barangays in Bolinao town, Pangasinan, Philippines	Consumed young leaf juice added with milk	Not applicable	Not applicable
Ethnobotanical information	Roldan Fiscal R (2017) [29]	Philippines	32 traditional healers in Laguna, Philippines	Consumed pounded pure leaf juice	Not applicable	Not applicable
Ethnobotanical information	Islam ATM et al. (2020) [27]	Bangladesh	41 elderly Rakhine tribes, including traditional health practitioners, in 2 districts of Bangladesh	Consumed pure leaf juice until recover	Not applicable	Not applicable
In vitro	Ranasinghe P et al. (2012) [30]	Sri Lanka	Heat-induced hemolysis erythrocytes obtained from healthy volunteers and dengue patients	Crushed, filtered, centrifuged and freeze-dried fresh leaf juice added with water (37.5 µg/mL)	Aspirin (90 µg/mL)	Higher inhibition on healthy and dengue infected erythrocytes Intervention (for all 3 leaf maturities) vs. control: X Higher inhibition on dengue infected erythrocytes Intervention (for partly mature leaves) vs. control: X
In vitro	(same as above)	Sri Lanka	Hypotonicity-induced hemolysis erythrocytes obtained from healthy volunteers and dengue patients	Crushed, filtered, centrifuged and freeze-dried fresh leaf juice added with water (37.5 µg/mL)	Indomethacin (No dose given)	Higher inhibition on healthy and dengue infected erythrocytes Intervention (for partly mature leaves) vs. control: X
In vivo	Dharmarathna SLCA et al. (2013) [44]	Sri Lanka	Male healthy white mice (32–33 g body weight, 18 mice per group)	Oral gavage once daily 0.2 mL of blended pure fresh leaf juice for 7 days and observed for extra 14 days	Oral gavage once daily water for 7 days and observed for extra 14 days	Increase platelet count Intervention vs. control: O Increase red blood cell count Intervention vs. control: O
In vivo	Rubio ICS (2016) [33]	Philippines	Mosquito larvae (5 larvae per treatment time) captured from artificial mosquitoes’ trap that contain only clear water (8-week exposed at outdoor)	Pounded and squeezed pure leaf juice (0.5 mL) for 5-, 20- and 35-min treatment	Not applicable	All the larvae died within the treatment time frames. Mosquitoes’ larvae trapped was belong to *Aedes* sp. and *Culex* sp.
In vitro	Chinnappan et al. (2016) [31]	India	Adenosine diphosphate-induced platelet aggregation on plasma rich platelet and plasma poor platelet obtained from 60 healthy volunteers and 60 dengue patients	Grinded, strained and freeze-dried mature fresh pure leaf juice (No dose given)	Untreated plasma platelet	Decrease platelet aggregation Intervention vs. control: O Intervention (pre-infected with intervention pre-treated dengue plasma poor platelet) vs. control: O
In vitro	Jayasinghe CD et al. (2017) [32]	Sri Lanka	Peritoneal macrophages isolated from healthy Wistar rats	Blended dried pure leaf juice (62.5, 125, 250, 500, 1000 µg/mL)	Complete RPMI 1640 cell media	Higher phagocytic activity Intervention vs. control: O Increase IFN-γ Intervention vs. control: O * Increase IL-10 Intervention vs. control: O *
Ex vivo	(same as above)	Sri Lanka	Bone marrow cells (absence of mitogen) and splenocytes isolated from healthy Wistar rats	Blended dried pure leaf juice (31.25, 62.5, 125, 250, 500, 1000 µg/mL)	Complete RPMI 1640 cell media	Higher proliferation activity of bone marrow cells Intervention (31.25 µg/mL) vs. control: O Higher proliferation activity of splenocytes Intervention (31.25 µg/mL) vs. control: O Increase IFN-γ from bone marrow cells Intervention (62.5–1000 µg/mL) vs. control: O Increase IFN-γ from splenocytes Intervention (31.25–1000 µg/mL) vs. control: O Increase IL-10 from bone marrow cells Intervention (62.5 µg/mL) vs. control: O Increase IL-10 from splenocytes Intervention (31.25–500 µg/mL) vs. control: O
In vivo	(same as above)	Sri Lanka	Healthy Wistar rats (both genders, 180–230 g body weight, 6 rats per group)	Oral gavage once daily 0.36 and 0.72 mL/100 g body weight of blended dried pure leaf juice for 3 days	Oral gavage once daily distilled water for 3 days	Increase platelet count Intervention vs. control: O * Increase bone marrow cells Intervention vs. control: O * Increase total white blood counts Intervention vs. control: O * Increase monocyte and lymphocyte count Intervention vs. control: O * Increase TNF-α Intervention vs. control: O * Increase IL-6 Intervention vs. control: O * Higher phagocytic activity Intervention vs. control: O *
In vivo	Akhter T et al. (2014) [35]	Bangladesh	Cyclophosphamide-induced thrombocytopenia Long Evans Norwegian rats (150–200 g body weight, 6 rats per group)	Oral gavage once daily 2 mL of blended pure fresh leaf juice for 3 days	Subcutaneous once daily 0.1 mL of hydrocortisone for 3 days	Increase platelet count Intervention vs. control: O
In vivo	Jayawardhane NDCKK (2014) [34]	Sri Lanka	Healthy adult Wistar rats (both genders, 180–250 g body weight, 6 rats per group)	Oral gavage once daily 0.72 mL/100 g body weight of blended pure mature leaf juice for 3 days	Oral gavage once daily distilled water for 3 days	Increase platelet count Intervention vs. control: O Increase monocyte and lymphocyte counts Intervention vs. control: O Increase bone marrow cells Intervention vs. control: O Higher phagocytic activity Intervention vs. control: O
In vivo	Tahir N et al. (2014) [39]	Pakistan	Carboplatin-induced myelosuppression adult Swiss mice (either gender, 35–45 g body weight, 11 mice per group)	Oral gavage once daily 5 and 10 mL/kg body weight of pounded and squeezed pure medium size leaf juice (respectively, male and female varieties) for 21 days	Oral gavage once daily distilled water for 21 days	Platelet count Male variety vs. female variety: X Increase platelet count Intervention vs. control: O *
In vivo	Anjum V et al. (2017) [38]	India	Cyclophosphamide-induced thrombocytopenia female albino Wistar rats (200–300 g body weight, 6 rats per group)	Oral gavage once daily 50 and 150 mg/kg body weight of freeze-dried ground fresh leaf juice added with distilled water for 14 days	Oral gavage once daily 0.8 mL of saline for 14 days	Increase platelet count Intervention vs. control: O Increase monocytes, basophils, eosinophils, lymphocytes and neutrophils Intervention vs. control: O Shorter bleeding time Intervention (50 mg/kg body weight) vs. control: O Shorter clotting time Intervention vs. control: O
In vivo	(same as above)	India	Cyclophosphamide-induced thrombocytopenia female Swiss albino mice (30–45 g body weight, 6 mice per group) (re-induced thrombocytopenia condition on Day 8, 9 and 10)	Oral gavage once daily 150 mg/kg body weight of freeze-dried ground fresh leaf juice added with distilled water for 3 days and observed for another 7 days	Untreated mice	Decrease inflammation Intervention vs. control: O Increase total leukocyte count Intervention vs. control: O Decrease phagocytic index Intervention vs. control: O Decrease mean antibody titre Intervention vs. control: O Decrease TNF-α Intervention vs. control: O
In vivo	Mohd Abd Razak MR et al. (2018) [40]	Malaysia	AG129 male mice inoculated intraperitoneal with 2 × 10^6^ PFU of New Guinea C strain-DENV-2 or plain media (20–27 g body weight; 5 mice per group)	Oral gavage once daily 1000 mg/kg body weight of freeze-dried powder of blended pure fresh leaf juice for 3 days	Oral gavage once daily distilled water for 3 days	Plasma antigen level Intervention vs. control: X Plasma viral RNA level Intervention vs. control: X
In vivo	Santosh Kumar M et al. (2018) [36]	India	Hydroxyurea-induced thrombocytopenia albino rats (either gender; 100–125 g body weight, 6 rats per group)	Oral gavage once daily 0.18 and 0.36 mL/100 g body weight of pounded and squeezed pure mature leaf juice for 5 days	Untreated rats	Increase red blood cell count Intervention vs. control: O Shorter bleeding time Intervention vs. control: O
In vivo	Mohd Abd Razak MR et al. (2019) [41]	Malaysia	AG129 male mice inoculated intraperitoneal with New Guinea C strain-DENV-2 (2 × 10^6^ PFU) or plain media (20–27 g body weight; 3 or 4 mice per group)	Oral gavage once daily 1000 mg/kg body weight of freeze-dried powder of blended pure fresh leaf juice for 3 days	Untreated mice	Increase 1 gene expression Intervention vs. control: O Decrease 3 gene expressions Intervention vs. control: O
In vivo	Norahmad NA et al. (2019) [42]	Malaysia	AG129 male mice inoculated intraperitoneal with New Guinea C strain-DENV-2 (2 × 10^6^ PFU) or plain media (20–27 g body weight; 5 mice per group)	Oral gavage once daily 1000 mg/kg body weight of freeze-dried powder of blended pure fresh leaf juice for 3 days	Untreated mice	Decrease 8 inflammatory cytokines and receptors (CCL6/MRP- 1, CCL8/MCP-2, CCL12/MCP-5, CCL17/TARC, IL1R1, IL1RN/IL1Ra, NAMPT/PBEF1, PF4/CXCL4) in the liver Intervention vs. control: O
In vivo	Mohd Abd Razak MR et al. (2021) [43]	Malaysia	AG129 male mice inoculated intraperitoneal with Malaysian clinical DENV-2 (DMOF015) (2 × 10^5^ PFU) or plain media (7–8 weeks old; 20–27 g body weight; 5 mice per group)	Oral gavage once daily 500 and 1000 mg/kg body weight of freeze-dried powder of blended pure fresh leaf juice for 3 days	Untreated mice	Increase total white blood cell count Intervention (1000 mg/kg body weight) vs. control: O Increase neutrophil count Intervention (1000 mg/kg body weight) vs control: O Decrease 5 plasma cytokines Intervention vs. control: O Decrease IL-6 in liver Intervention (500 mg/kg body weight) vs. control: O Decrease viral RNA in liver Intervention vs. control: O
In vivo	Nandini C et al. (2021) [37]	India	Cyclophosphamide-induced thrombocytopenia Sprague Dawley rats (180–200 g body weight, 8 rats per group)	Oral gavage once daily 200 and 400 mg/kg body weight of freeze-dried blended and squeezed pure fresh leaf juice for 14 days	Untreated rats; Oral gavage once daily water for 14 days	Shorter bleeding time Intervention vs. untreated control: O * Shorter clotting time Intervention vs. untreated control: O * Decrease cellular malondialdehyde Intervention vs. untreated control: O * Decrease serum thrombopoietin cytokine Intervention vs. untreated control: O * Increase SOD Intervention vs. untreated control: O * Increase GSH Intervention vs. untreated control: O * Increase platelet count Intervention vs. water control: O * Shorter prothrombin time Intervention vs. untreated control: O Increase MPL-CD110 Intervention vs. untreated control: O
Human (case report)	Ahmad N et al. (2011) [46]	Pakistan	A 45 year old male dengue patient treated with standard treatment for first 5 days (different broad spectrum of antibiotics, anti-malarial drugs)	Consumed twice daily (in the morning and evening) 25 mL of ground leaf juice added with water and sucrose for next 5 days	Not applicable	Increase platelet count. Increase level of white blood cell. Increase level of neutrophil.
Human (case report)	Deepak BSR et al. (2013) [47]	India	A 51 year old male dengue fever patient treated with standard treatment (ringer lactate, dexamethasone, gramocef, paracetamol)	Consumed twice daily 25 mL of ground tender leaf juice added with water for 8 days (first 5 days together with standard treatment, next 3 days together with Ayurveda treatment)	Not applicable	Increase platelet count. Increase white blood cell counts. Patient discharged on a day after completing intervention treatment period.
Human (case report)	Siddique O et al. (2014) [45]	Pakistan	A 23 year old male dengue patient treated with azithromycin 250 mg once daily, acetaminophen per 8-h, unlimited amount of oral hydration for the first 5 days	Consumed once daily 150 mL of ground leaf juice added with water and took alternate sips between intervention and commercially-made fruit juice for next 5 days	Different days of treatment	Increase platelet count. Increase level of white blood cell. Increase level of hemoglobin.
Human (cross-sectional)	Ismail IS et al. (2019) [48]	Malaysia	Dengue patients admitted to Hospital Universiti Sains Malaysia Kelantan between January 2014 and December 2015 (≥18 years old, 214 respondents) treated with standard treatment	Consumed at least once daily leaf juice for 3 days	Not applicable	131 out of 214 respondents
Human (quasi trial)	Hettige S (2008) [49]	Sri Lanka	Dengue patients (6 females and 6 males, adult and children (<10 years old), 4 children and 8 adults) also received standard oral treatment (antiemetic, paracetamol, antibiotics) as necessary	Consumed twice in a day (8-h interval) of crushed and squeezed pure tender fresh leaf juice (2 leaves) for 1 day (adult: 5 mL, children: 2.5 mL)	Before/after treatment	Increase white blood cell After vs. before: O Increase platelet count After vs. before: O 5 patients no longer experienced hemorrhagic skin rash. All 12 patients recovered with no hospital admission.
Human (quasi trial)	Naresh Kumar CVM et al. (2015) [52]	India	Dengue patients (6 females and 3 males) received usual management (saline, anti-emetics, paracetamol) as necessary (only after receiving intervention treatment)	Consumed thrice daily (6-h interval) 5 mL of blended and filtered fresh partly mature leaf juice added with sucrose for 6 days	Different days of treatment	Increase total white blood cell Day 3 vs. day 1, day 2: O Day 6 vs. day 1, day 2, day 3, day 4, day 5: O Increase platelet count Day 3 vs. day 1, day 2: O Day 6 vs. day 1, day 2, day 3, day 4, day 5: O All 9 patients gradually recovered from lethargy, fatigue, and fever. No excess fluid collected at pleural, pericardial and peritoneal sites of patients after intervention treatment.
Human (quasi trial)	Prakash Kala C (2012) [50]	India	Dengue patients (19–52 years old, 5 subjects)	Consumed thrice daily (6-h interval) 2 tablespoons of crushed, squeezed and filtered pure fresh leaf juice (2 leaves) for 1 day	Before/after treatment	Increase platelet count After vs. before: O
Human (quasi trial)	Solanki SG et al. (2020) [51]	India	Dengue patients 100 patients in intervention group (42 females and 58 males), 50 patients in control group (20 females and 30 males)	Consumed thrice daily of blended fresh leaf juice added with water (adult: 10 mL, children: 5 mL) for 3 days (together with 1 kiwi fruit per consumption)	Before/after treatment	Increase white blood cell After vs. before: O Increase platelet count After vs. before: O
Human (open labelled RCT)	Subenthiran S et al. (2013) [53]	Malaysia	Dengue patients (18–60 years old), grade 1 and 2 dengue fever, 111 patients in intervention group (20 females and 91 males), 117 patients in control group (14 females and 103 males))	Consumed once daily 30 mL of blended pure leaf juice for 3 days (together with standard treatment)	Standard treatment	Increase platelet count Intervention 40-h vs. 8-h: O Control 48-h vs. 8-h: O
Human (open labelled RCT)	Hettige S et al. (2020) [54]	Sri Lanka	Dengue patients (16–60 years old), 43 subjects in intervention group (7 females and 36 males), 76 subjects in control group (15 females and 61 males) who have at least seven days of fever but not dengue hemorrhagic fever	Consumed twice daily (12-h interval) 20 mL of blended mature leaf juice added with water until the day of discharge	Standard treatment	Shorter total duration of illness Intervention vs. control: O Shorter duration of fever Intervention vs. control: O Shorter duration of hospitalization Intervention vs. control: O Lesser episode of pleural effusion Intervention vs. control: O

RPMI: Roswell Park Memorial Institute medium; IFN: interferon; IL: interleukin; TNF: tumor necrosis factor; SOD: superoxide dismutase; GSH: glutathione; MPL-CD110: thrombopoietin receptor; AG129: mouse deficient in IFN-α, β, γ receptor signaling; PFU: plaque forming unit; DENV-2: dengue virus serotype 2; RNA: ribonucleic acid; CCL: chemokine (c-c motif) ligand; MRP: multi drug resistance-associated protein; MCP: monocycte chemoattractant protein; TARC: thymus and activation-regulated chemokine; IL1R: interleukin-1 receptor; IL1RN: interleukin-1 receptor antagonist; NAMPT/PBEF1, PF4: platelet factor 4; CXCL4: chemokine (c-x-c motif) ligand; X: not significant (*p* > 0.05); O: significant (*p* < 0.05); *: dose-dependent manner.

**Table 3:** Summary of juice preparations reported in the included studies.

Author (Year)	Ingredient Added into Juice	Leaf Maturity	Leaf Condition	Leaf Cleansing	Juice Extraction Technique
Hettige S (2008) [49]	None	Tender	Fresh	Not mentioned	Crush, squeeze
Ahmad N et al. (2011) [46]	Water and sucrose	Not mentioned	Not mentioned	Rinse with water	Grind
Prakash Kala C (2012) [50]	None	Not mentioned	Fresh	Rinse with water	Crush, squeeze, filter
Ranasinghe P et al. (2012) [30]	Water	Immature, partly mature, mature	Fresh	Rinse with water	Crush, filter, centrifuge, freeze-dry
Deepak BSR et al. (2013) [47]	Water	Tender	Not mentioned	Rinse with water	Grind
Dharmarathna SLCA et al. (2013) [44]	None	Middle stage age	Fresh	Rinse with water; remove stems	Blend
Subenthiran S et al. (2013) [53]	None	Not mentioned	Not mentioned	Rinse with water	Blend
Akhter T et al. (2014) [35]	None	Not mentioned	Fresh	Remove petioles and veins	Blend
Jayawardhane NDCKK (2014) [34]	None	Mature	Not mentioned	Rinse with water; remove petioles, primary veins and leaf blades	Blend
Siddique O et al. (2014) [45]	Water	Not mentioned	Not mentioned	Rinse with water	Grind
Tahir N et al. (2014) [39]	None	Medium size	Not mentioned	Rinse with water	Pound, squeeze
Chinnappan et al. (2016) [31]	None	Mature	Fresh	Rinse with water; remove petioles, veins and leaf blades	Grind, strain, freeze-dry
Rubio ICS (2016) [33]	None	Not mentioned	Not mentioned	Not mentioned	Pound, squeeze
Anjum V et al. (2017) [38]	Water	Not mentioned	Fresh	Remove woody stalks	Chop, grind, filter, freeze-dry
Fajardo WT et al. (2017) [28]	Milk	Young	Not mentioned	Not mentioned	Not mentioned
Jayasinghe CD et al. (2017) [32]	None	Mature	Dry	Rinse with water; remove petioles and primary veins	Blend
Roldan Fiscal R 2017 [29]	None	Not mentioned	Not mentioned	Not mentioned	Pound
Mohd Abd Razak MR et al. (2018) [40]	None	Not mentioned	Healthy (without ring spot)	Rinse with water and veggie wash	Blend, freeze-dry
Santosh Kumar M et al. (2018) [36]	None	Mature	Not mentioned	Rinse with water; remove petioles, primary veins and leaf blades	Crush, pound, squeeze
Ismail IS et al. (2019) [48]	Not mentioned	Not mentioned	Not mentioned	Not mentioned	Not mentioned
Mohd Abd Razak MR et al. (2019) [41]	None	Not mentioned	Healthy (without ring spot)	Rinse with water and veggie wash	Blend, freeze-dry
Norahmad NA et al. (2019) [42]	None	Not mentioned	Healthy (without ring spot)	Rinse with water and veggie wash	Blend, freeze-dry
Hettige S et al. (2020) [54]	Water	Mature	Not mentioned	Not mentioned	Grind, blend
Islam ATM et al. (2020) [27]	Not mentioned	Not mentioned	Not mentioned	Not mentioned	Not mentioned
Solanki SG et al. (2020) [51]	Water	Not mentioned	Fresh	Not mentioned	Blend
Mohd Abd Razak MR et al. (2021) [43]	None	Not mentioned	Healthy (without ring spot)	Rinse with water and veggie wash	Blend, freeze-dry

Definitions for terminologies used to describe the leaf maturity in the original article were not given.

**Table 4:** Summary of chemical compositions identified in *Carica papaya* leaf juice by 10 included studies.

Author (Year)	Chemical Composition
Ranasinghe P et al. (2012) [30]	Phenolics; flavonoids
Subenthiran S et al. (2013) [53]	Manghaslin; clitorin; rutin
Jayawardhane NDCKK (2014) [34]	Polyphenols; flavonoids; tannins; saponins; alkaloids; carbohydrates; proteins; amino acids
Anjum V et al. (2017) [38]	Myricetin; caffeic acid; trans-ferulic acid; kaempferol
Jayasinghe CD et al. (2017) [32]	Phenolics; flavonoids; bis (2-(2-chloroethoxy)ethyl) ether; dimethoxydimethylsilane; 3-benzoyl-8-oxo-6-azabicyclo [3.2.1] octan-6,7-dicarboxylicacid, dibenzyl ester; benzhydrazide; o-butylisourea; 10-oxatetracyclo [5.5.2.0(1,5).0(8,12)] tetradecene-9,11,14-trione; 4-[(2-methoxyethoxy)methoxyl]- 5-methyl-; 2-chloro-5,5-dimethyl-1-phenyl-3- hexen-1-ol; 2-methoxybenzeneacetaldehyde; 1-methyl-2-pyrrolidinone; benzonitrile; nonanal; octanoic acid; methyl ester; 1-decene, n-benzyl-n-phenylethylisobutyramide; nonanoic acid; benzene; 1,3-bis(1,1-dimethylethyl)-; 1-iodooctadecane; 2-methylnaphthalene; 2-tetradecene; 10-undecenoic acid; dodecanal; 1,4-dimethylnaphthalene; 9-oxononanoic acid; 1-hentriacontane; 2,4-di-tert-butylphenol; nonanedioic acid; dimethyl ester; azelaic acid; 2-tetradecene; 1-octadecene; 1-hexadecanoic acid; n-hexadecanoic acid; cycloeicosane; 9-octadecenoic acid; cethyl stearate; methyl 2-octylcyclopropene-1- heptanoate; 9,12-octadecadienoic acid
Mohd Abd Razak MR et al. (2018) [40]	Quinic acid; malic acid; protocatechuic acid; chlorogenic acid; *p*-coumaric; caffeic acid; manghaslin; clitorin; sinapic acid; isoquercetin; ferulic acid; rutin; astragalin; nicotiflorin; deoxyhydrocarpaine I; deoxyhydrocarpaine II; myricetin; fisetin; morin; quercetin; kaempferol; citropten; isorhamnetin
Norahmad NA et al. (2019) [42]	Manghaslin; clitorin; rutin; nicotiflorin
Nandini C et al. (2021) [37]	Benzoic acid; *o*-methyl syringic acid; caffeic acid; syringic acid; gallic acid; ferulic acid; veratric acid; 3,4,5-trihydroxy cinnamic acid; kaempferol; dimethyl caffeic acid; protocatechuic acid; quercetin; 4-hydroxy; trans-cinnamic acid; carpaine
Mohd Abd Razak MR et al. (2021) [43]	Manghaslin; clitorin; rutin; nicotiflorin; carpaine

Chemical nomenclature used above are solely based on the original article.

## Data Availability

Not applicable.
